# Successful Outcome of Triangle Tilt as Revision Surgery in a Pediatric Obstetric Brachial Plexus Patient with Multiple Previous Operations

**DOI:** 10.1155/2014/715389

**Published:** 2014-11-23

**Authors:** Rahul K. Nath, Vishnu Halthore, Chandra Somasundaram

**Affiliations:** Texas Nerve and Paralysis Institute, 6400 Fannin Street, Houston, TX 77030, USA

## Abstract

*Introduction*. Obstetric brachial plexus injury (OBPI) occurs during the process of labor and childbirth. OBPI has been reported to be associated with shoulder dystocia, macrosomia, and breech delivery. Its occurrence in uncomplicated delivery is possible as well. *Case Presentation*. The patient in the present report is a 6.5-year-old girl, who suffered a severe brachial plexus injury at birth and had many reconstructive surgical procedures at an outside brachial plexus center before presenting to us. *Discussion*. The traditional surgical treatments by other surgical groups were unsuccessful and therefore the patient came to our clinic for further treatment. She had triangle tilt surgery with us, as a salvage procedure. *Conclusion*. The OBPI patient in this study clearly showed noticeable clinical and functional improvements after triangle tilt surgical management. The posture of the arm at rest was greatly improved to a more normal position, and hand to mouth movement was improved as well. Triangle tilt surgery should be conducted as a first choice treatment for medial rotation contracture of the shoulder in OBPI patients.

## 1. Introduction

Obstetric brachial plexus injury (OBPI) occurs during the process of labor and childbirth. OBPI has been reported to be associated with shoulder dystocia, [1–3] macrosomia [3, 4], and breech delivery [5]. Its occurrence in uncomplicated delivery is not uncommon [6–8]. Most injured infants recover upper extremity function spontaneously [9, 10]. However, roughly one in 5 affected infants tends to retain persistent limb deficits, never recover full function, and develop permanent injuries [11]. The most commonly affected nerve roots of the brachial plexus are C5-6, with or without injury to the other roots, affecting any or all of the C5-T1 nerve roots. C8-T1 roots are rarely affected [12]. Persistent C5 nerve damage in these patients leads to muscle imbalances and bony deformities at the shoulder joint. Significant shoulder secondary deformities that follow include medial rotation contracture and elbow flexion contracture [12].

Many traditional procedures have been described to treat obstetric brachial plexus deformity [13–16]. Here, we report the clinical outcome of one OBPI pediatric patient, who had undergone the triangle tilt surgical procedure as a salvage procedure at our institution [17–24].

## 2. The Triangle Tilt Surgical Procedure

The triangle tilt is an established novel osseous surgical procedure, consisting of osteotomy of the clavicle at the junction of the middle and outer thirds, osteotomy of the acromion at its junction with the spine of the scapula, ostectomy of the superomedial angle of the scapula, anterior shoulder capsule, and soft tissue releases and splinting of the limb in adduction, 5° of external rotation, and full forearm supination [17–24]. These individual procedures together release the distal acromioclavicular triangle and allow it to reorient itself in a more neutral position into the glenoid fossa. Further, this operative technique addresses the SHEAR [25] deformity and its central influence in the pathophysiology of the medial rotation contracture (MRC) and the shoulder deformity.

## 3. Case Presentation

**Figure 1:**
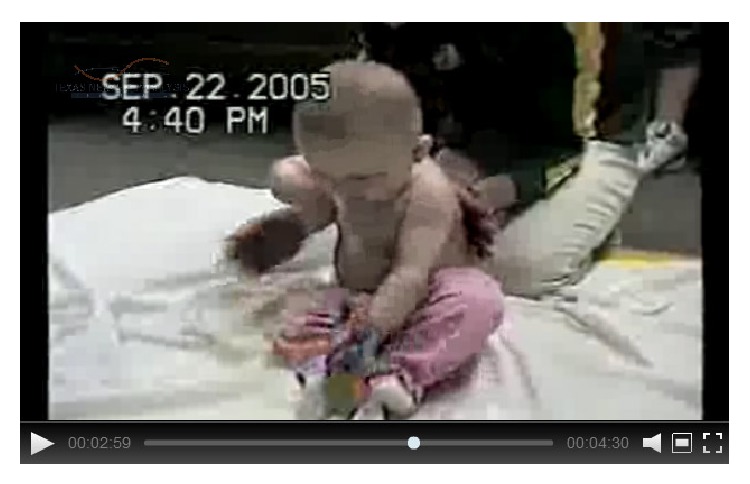
The patient (8 months old) with severe obstetric brachial plexus injury with bent elbow, shorter arm with internal rotation of the shoulder.

The patient in the present report is a 6.5-year-old girl who was born with right side brachial plexus injury (C5–C8), weighing 9 lb 13 oz. She did not have a documented history of shoulder dystocia and was delivered normally without the use of any instruments. There was no finger movement at birth. The patient initially presented to us when she was 8 months old with severe obstetric brachial plexus injury (Figure 1). She had a flexed elbow, a shorter arm with medial rotation of the shoulder. We recommended triangle tilt surgery for the medial rotation of the shoulder with contracture and flexed elbow. Nerve transfer or nerve graft surgeries were not recommended [26]. Instead, the child was referred to another surgeon by her insurance company for treatment, where she underwent the following operations over 5+ years' time:

1. brachial plexus decompression,
2. shoulder joint reduction,
3. decompression of subacromial space,
4. subscapularis lengthening,
5. glenohumeral joint ligament releases,
6. latissimus dorsi tendon transfer,
7. teres major tendon transfer,
8. end-to-side axillary nerve to triceps nerve transfer,
9. end-to-side axillary nerve to radial nerve transfer,
10. derotational humeral osteotomy,
11. biceps to triceps tendon transfer using cadaver donor graft,
12. elbow lengthening with cadaver donor graft.

**Figure 2:**
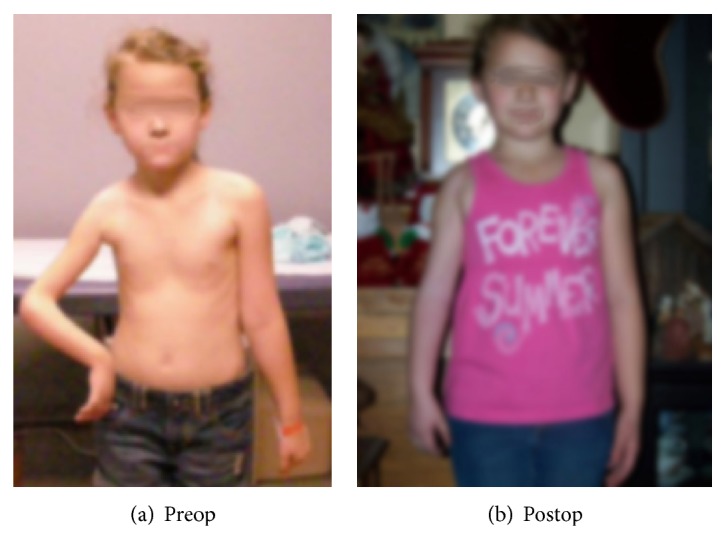
Clinical photograph of the patient after multiple surgeries at an outside hospital but before triangle tilt surgery (a) and 2 months after triangle tilt surgery (b), showing significant improvement in resting arm posture.

**Figure 3:**
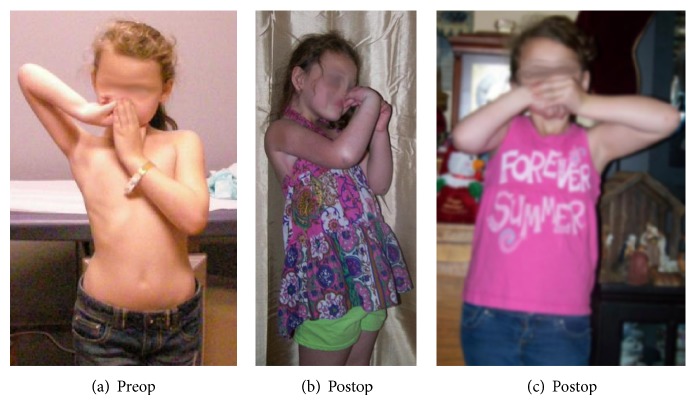
Functional movement picture of the patient after multiple surgeries at an outside hospital but before triangle tilt surgery (a) and 2 months after triangle tilt surgery (b), showing improvement in hand to mouth movement.

**Figure 4:**
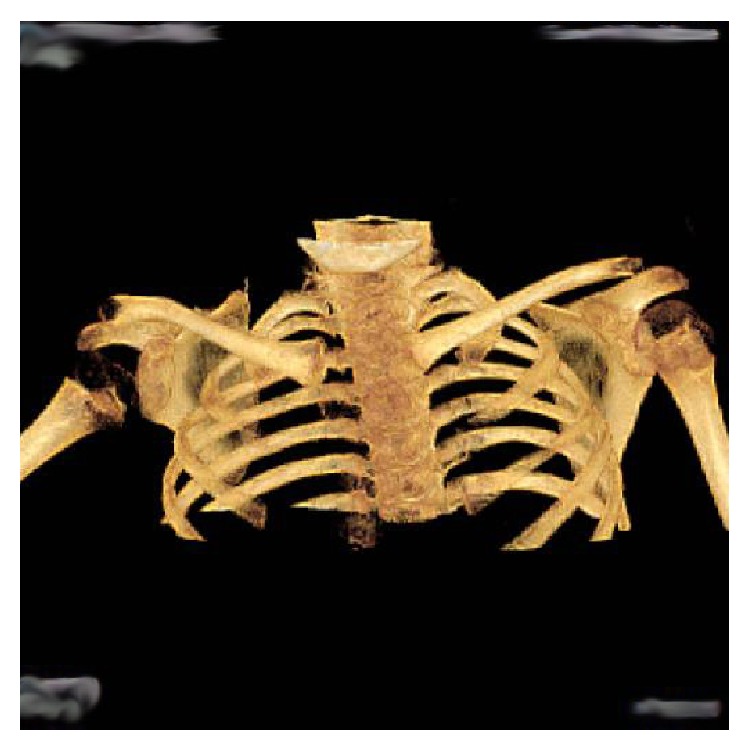
Radiological picture of the patient after multiple surgeries at an outside hospital but before triangle tilt surgery, showing SHEAR deformity [25].

This patient had persistent internal rotation deformity (among many other ongoing problems) related, by preoperative clinical examination and radiological studies, to an elevated scapula with forward rotation (Figure 4), the SHEAR deformity [25]. This placed the humerus into an internally rotated position in the glenohumeral joint and, therefore, resulted in the abnormal posture prior to surgery. The patient's findings specifically indicated treatment with triangle tilt surgery [17–24] at our clinic. The senior author (RKN) performed the surgical procedures. The post-triangle tilt pictures of the patient are presented in Figures 2(b), 3(b), and 3(c).

Procedures performed on this patient are as follows:

1. scapular ostectomy,
2. spinous process/acromion process osteotomy,
3. bone graft; acromion process,
4. anterior glenohumeral capsule release and subscapularis tendon release,
5. pectoralis major and minor release,
6. subscapularis tendon transfer,
7. clavicle osteotomy.

## 4. Discussion

The outcome of all the above surgeries at the outside institution was poor. There was no change in the clinical picture and the patient had significant functional issues, with no improvement in physical deformities (Figures 2(a) and 3(a)). The use of cadaver donor graft may have complicated the surgical outcome. A published study has shown that cadaver tissue failed in 23% of patients younger than 40 in ligament reconstructive surgeries [27], questioning their reliability in children. Further, the potential for communicable disease transmission in a young child is concerning. The child was discharged from care at the age of 6.5 years from the outside institution and eventually came to us for treatment.

Two months following triangle tilt surgery, initial post-SARO images showed neutral position of the affected shoulder and significant improvement in overall arm and shoulder position. The shoulder appeared congruent and the arm was straight (Figure 2(b)). The girl was able to perform much improved hand to mouth movements postoperatively (Figures 3(b) and 3(c)). We do not have follow-up pictures for other functional movements.

## 5. Conclusion

The OBPI patient in this study clearly showed noticeable clinical and functional improvements after triangle tilt surgical management. The posture of the arm at rest was greatly improved to a more normal position, and hand to mouth movement was improved as well. Triangle tilt surgery should be conducted as a first choice treatment for medial rotation contracture of the shoulder in OBPI patients.
